# ACEing premature codon termination using anticodon-engineered sup-tRNA-based therapy

**DOI:** 10.1016/j.omtn.2022.07.019

**Published:** 2022-08-06

**Authors:** Ting-Yu Lin, Sebastian Glatt

**Affiliations:** 1 Malopolska Centre of Biotechnology (MCB), Jagiellonian University, 30-387 Krakow, Poland

A nonsense mutation is a single genomic nucleotide substitution that converts an amino acid coding triplet of a mRNA into a translation termination signal, also known as stop codons (TGA, TAG, or TAA). If a nonsense mutation happens in a frame of a coding region, it causes premature codon termination (PTC) during protein synthesis, without the possibility of rescuing the ribosome machinery via readthrough. This chain of events results in the production of non-functional truncated proteins that lack its genuine activity, and the truncated polypeptide can be additionally harmful to cells. Furthermore, nonsense-mediated decay (NMD) degrades the PTC-containing mRNA transcripts, making it more deleterious than other missense mutation. Nonsense mutations account for 10%–15% of all severe genetic diseases, including cystic fibrosis, Duchenne muscular dystrophy, and spinal muscular atrophy.^1^

Efforts to identify PTC therapeutics have focused on restoring full-length protein levels by gene therapy and nonsense suppression. Small-molecule readthrough agents, such as gentamicin or ataluren, bind to ribosomes to globally promote readthrough but also have severe drawbacks such as reduced global translational fidelity or high nephron- and ototoxicity.^2^ Gene therapy approaches by CRISPR-Cas gene editing can correct the faulty genes but require a tailored solution for each individual mutation and carry the risk of off-target editing events. Therapeutic suppressor tRNAs (sup-tRNA) provide a rediscovered therapeutic alternative, which was first proposed three decades ago and was initially employed in a β-thalassemia model. Anticodon-engineered sup-tRNA (ACE-tRNA) technology could offer a generalizable solution for a broad array of PTC-associated diseases.^3^ ACE-tRNAs carry an altered anti-codon sequence that recognizes a nonsense mutation while being charged with correct amino acids by endogenous aminoacyl-tRNA synthetases. Nonetheless, most available reports only demonstrate the rescue of protein expression by sup-tRNAs in prokaryotic and yeast model systems. Therefore, aspects of biosafety, bioavailability, and the efficacy of ACE-tRNAs are yet to be determined in mammalian systems and leave fundamental questions open—for instance, whether genuine PTCs in their genomic context have an impact on the efficacy of suppressing tRNA rescue.

**Figure 1:**
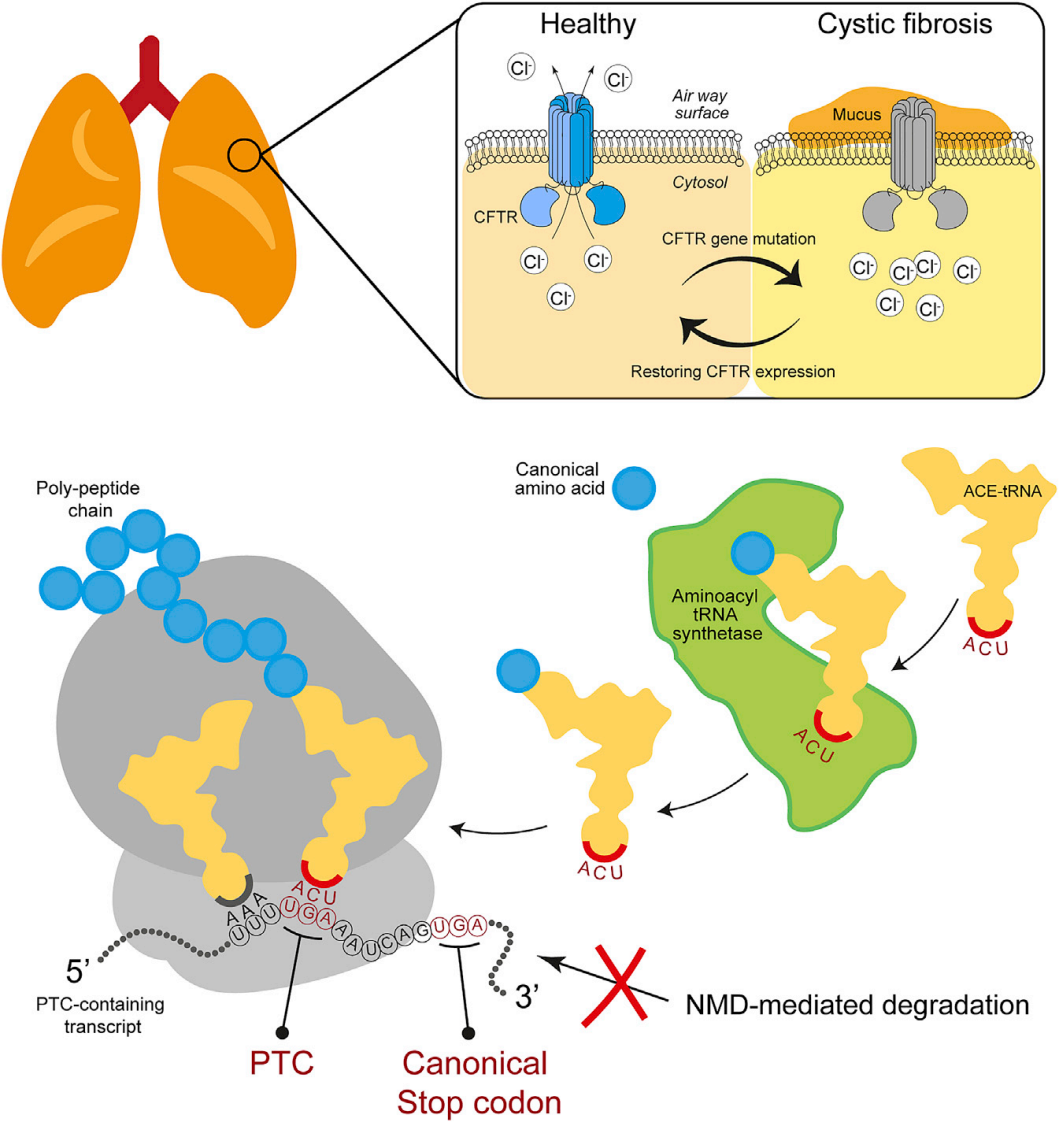
Application of ACE-tRNAs as a therapeutic to PTC-causing-CFTR treatment (Top) A scheme of epithelial lung cells with CFTR locating at cell member for pumping chloride (left), while loss of CFTR (shown in gray) causes reduction in airway clearance and accumulating mucus. (Bottom) Mechanism of ACE-tRNA promoting PTC readthrough. The ACE-tRNA is charged with a canonical amino acid by the endogenous aminoacyl tRNA synthetase. The correctly charged ACE-tRNA recognize the PTC and incorporates the amino acid to the poly-peptide chain during ribosome-mediated translation process. NMD-mediated mRNA degradation is inhibited by ACE-tRNA treatment.

In this issue of *Molecular Therapy – Nucleic Acid*, Ko et al. used an ACE-tRNA library to confirm the effectiveness of ACE-tRNAs on suppressing the endogenous PTC-containing mRNAs in human cell lines and disease models.^4^ As a reported system, they integrated a PTC-containing NanoLuc reporter gene into genomic TTAA sites using the *piggyBac* transposon/transposase system. In a pilot experiment, the reporter system allowed them to directly identify the best-performing ACE-tRNAs. Their study specifically targets the cystic fibrosis transmembrane regulator (CFTR) gene that accounts for 10% of all mutations causing cystic fibrosis. They used the 16HBE14o-, human bronchial epithelial cell line, and the derivative 16HBE-gene-edited (16HBEge) cell lines carrying engineered R1162X-, W1282X-, or G542X-CFTR variants. To rescue the PTC-mediated protein loss, the authors provided appropriate ACE-tRNAs including ACE-tRNA^Arg^, ACE-tRNA^Gly^, and ACE-tRNA^Leu^ and introduced these molecules into cells using transient transfection and a stably integrated reporter. For the W1282X variant, ACE-tRNA^Trp^ was not used as it possesses low nonsense-suppression activity, but ACE-tRNA^Leu^ can be substituted due to its robust restoring activity (see Figure 1).

Ko et al. further demonstrate the rescue of CFTR mRNA expression in 16HBEge cells. They transiently treated the cells with increasing amounts of ACE-tRNAs and showed that multiple ACE-tRNAs within each delivered vector indeed stabilized the mRNA transcripts—restoring levels up to 40%–50% of wild type. In addition, delivery of ACE-tRNA^Arg^ as RNA results in a similar mRNA rescue efficacy (32% of wild type), which peaks shortly (5 h) after delivery. However, this effect drops sharply 1 day after transfection due to the short half-life time of ACE-tRNAs. Moreover, the authors observed that the inhibiting NMD efficacy is positively correlated to the efficiency of tRNA delivery via both routes, namely via cDNA or RNA.

In connection with the stabilization of mRNA transcripts, Ko and colleagues provide further evidence of successful expression of full-length CFTR protein. Furthermore, the expressed CFTR protein from R1162X and W1282X-CFTR 16HBEge cells is correctly trafficked to cell membranes and is fully glycosylated. To assess the ACE-tRNA-dependent restoration of CFTR channel activity, the authors had to use a stable integrated ACE-tRNA expression system. Cells require a minimum 4-day cell culture to form tight junctions. The R1162X-CFTR 16HBEge cells was stably integrated with 16 copies of the ACE-tRNA^Arg^ transposon. Neither cellular morphology nor cell division rates were changed after 20 passages compared with the parental cell lines. This suggests that the expression of ACE-tRNAs is nontoxic, which is important when considering possible future clinical applications. The authors also showed that only 16 ACE-tRNA^Arg^ expression cassettes are integrated per genome. Remarkably, even the relatively low number of genomic copies leads to a moderate increase in CFTR mRNA and significantly enhances decoding of PTC to promote CFTR channel function in electrophysiological assays—almost to wild-type levels. To this end, intensifying the efforts to comprehensively sample all CF-causing PTCs will be a clear future goal of the group.

The authors’ findings provide strong evidence that the use of ACE-tRNAs seems to be a feasible approach to treat PTCs. The reported systems are shown to be compatible with different cell lines and the use of ACE-tRNAs not only obviates NMD-mediated mRNA transcript degradation but also increases PTC-containing transcript readthrough. More importantly, the persistence expression of ACE-tRNAs does not seem to cause any severe cellular side effects, like cytotoxicity, which is in stark contrast to the known G418 treatment. Further studies should address how to overcome the delivery efficiency hurdle in animal disease models, as efficient delivery systems for tRNAs are urgently needed. Since Ko et al. have demonstrated that ACE-tRNAs can work efficiently when delivered as cDNA or RNA, adeno-associated-virus- or nanoparticle-mediated delivery hold great potential.^5^ From a translational perspective, the next step is to leverage animal models of PTC-causing CF to determine the required minimum tRNA copies that should be delivered to restore CFTR activity in affected tissues. With this in mind, Ko et al. have shown that a relatively low amount of ACE-tRNA expression cassettes are required to restore full protein activity and cellular functions, which could lower the required therapeutic delivery efficiency. An exciting period of research lies ahead, which should see further development of ACE-tRNAs therapeutics—in particular, for treatment of nonsense mutation-associated diseases.
